# Mid-Term Results of Surgical Treatment of Atrial Fibrillation in Valvular Heart Disease Assesed by Speckle Tracking Echocardiography

**DOI:** 10.5935/abc.20180040

**Published:** 2018-04

**Authors:** Natalia Lorenzo, Irene Mendez, Mikel Taibo, Gianfranco Martinis, Sara Badia, Guillermo Reyes, Rio Aguilar

**Affiliations:** 1 Hospital Universitario Infanta Cristina, Parla, Madrid - Spain; 2 Hospital Universitario de La Princesa, Madrid - Spain

**Keywords:** Ablation Techniques, Atrial Fibrillation, Heart Valve Diseases, Cryosurgery, Echocardiography

## Abstract

**Background:**

Atrial fibrillation frequently affects patients with valvular heart disease. Ablation of atrial fibrillation during valvular surgery is an alternative for restoring sinus rhythm.

**Objectives:**

This study aimed to evaluate mid-term results of successful atrial fibrillation surgical ablation during valvular heart disease surgery, to explore left atrium post-ablation mechanics and to identify predictors of recurrence.

**Methods:**

Fifty-three consecutive candidates were included. Eligibility criteria for ablation included persistent atrial fibrillation <10 years and left atrium diameter < 6.0 cm. Three months after surgery, echocardiogram, 24-hour Holter monitoring and electrocardiograms were performed in all candidates who maintained sinus rhythm (44 patients). Echo-study included left atrial deformation parameters (strain and strain rate), using 2-dimensional speckle-tracking echocardiography. Simultaneously, 30 healthy individuals (controls) were analyzed with the same protocol for left atrial performance. Significance was considered with a P value of < 0.05.

**Results:**

After a mean follow up of 17 ± 2 months, 13 new post-operative cases of recurrent atrial fibrillation were identified. A total of 1,245 left atrial segments were analysed. Left atrium was severely dilated in the post-surgery group and, mechanical properties of left atrium did not recover after surgery when compared with normal values. Left atrial volume (≥ 64 mL/m^2^) was the only independent predictor of atrial fibrillation recurrence (p = 0.03).

**Conclusions:**

Left atrial volume was larger in patients with atrial fibrillation recurrence and emerges as the main predictor of recurrences, thereby improving the selection of candidates for this therapy; however, no differences were found regarding myocardial deformation parameters. Despite electrical maintenance of sinus rhythm, left atrium mechanics did not recover after atrial fibrillation ablation performed during valvular heart disease surgery.

## Introduction

Atrial fibrillation (AF) is a serious and frequent problem in valvular heart disease (VHD) affecting more than 30% of these patients. VHD leads to pressure and/or volume overload of the atria, especially in the left atrium (LA) in left-sided disease. AF is associated with higher morbidity and mortality in general population, but even more in VHD patients, requiring low threshold of anticoagulation because of higher risk of thromboembolism. AF also affects the decision making for selection of prosthesis type.^1,2^

AF ablation during cardiac surgery has been demonstrated as a safe and effective procedure restoring sinus rhythm (SR). Although the original Cox-Maze procedure was described in patients with lone AF, its use has expanded to patients with associated organic heart disease.^3^ According to some authors, success rates of the procedure can exceed 80%. However, there are few data on the results of this technique in valvular patients with persistent AF.^4,5^

Myocardial strain and strain rate (strainR) represent the magnitude and rate, respectively, of myocardial deformation. Both atrial strain and strainR, obtained using either Doppler tissue imaging (DTI) or two-dimensional speckle-tracking echocardiography, have proved to be feasible and reproducible techniques to evaluate LA mechanics.^6^

The aims of this study were to evaluate mid-term results after successful surgical ablation (SA) of AF in VHD patients, to explore LA mechanics using ultrasound strain and strainR imaging after SA of AF during VHD surgery and to identify clinical and echocardiographic predictors of recurrence during follow-up.

## Methods

### Patient eligibility

We prospectively included candidates to surgical ablation, who underwent valvular heart surgery between May 2008 and May 2012 in our institution.

Patient eligibility criteria for AF surgery included: persistent AF of less than ten years of evolution and left atrial anteroposterior (AP) diameter at preoperative transthoracic echocardiogram in long axis view of less than 6.0 cm.^3,7^ All candidates were adequately informed and signed informed consent form for the procedure, according to the local ethics committee.

Fifty-three consecutive candidates who underwent valvular heart surgery were included to surgical ablation.

Success of AF ablation procedure was considered when patients maintained SR at the time of discharge. All these patients were selected for initial follow up. After rhythm stabilization, which is considered to occur at least 3 months after surgery,^8^ an echocardiogram was scheduled, and ambulatory 24 hour Holter monitoring and electrocardiograms were systematically performed in all candidates who remained in SR (44 patients). Holter monitoring was programmed one month after the echocardiographic study, and electrocardiograms were made during clinical visits (at least two visits during the first year of follow up). Patients with persistent AF during the first 3 months after surgery were excluded from the follow-up.

### Surgical technique

All the procedures were carried out by full sternotomy and extracorporeal circulation.

Surgical technique for cryoablation was the same as previously described.^9^ After aortic clamping, LA was opened when needed and left atrial appendage was ligated from its inside using a 3.0 monofilament suture. The cryoablation probe was placed for 60 s at a temperature between -100ºC and -160ºC. Lines were created surrounding pulmonary veins and also joining between these circles. Three more lines were performed: between the left pulmonary veins and the left appendage, between the left pulmonary veins and the P3 portion of the mitral annulus and between the tricuspid septal valve and the inferior cava.

In cases where left atriotomy was not needed (in isolated aortic interventions), high-intensity-focused-ultrasound (HIFU - Epicor) cardiac ablation was used. Epicor Medical Cardiac Ablation System (St Jude) is designed to deliver HIFU via an entirely epicardial approach and consists of an array of transducers positioned after proper sizing around the LA wall of the pulmonary vein orifices.^4^

### Echocardiographic study

A Vivid 7 Dimension ultrasound system (GE Healthcare) was used for the transthoracic echocardio graphic examination. All images and measurements were acquired with a MS4 matrix probe using the standard views according to the standards of the European Association of Echocardiography and the American Society of Echocardiography.^10,11^

**Figure 1:**
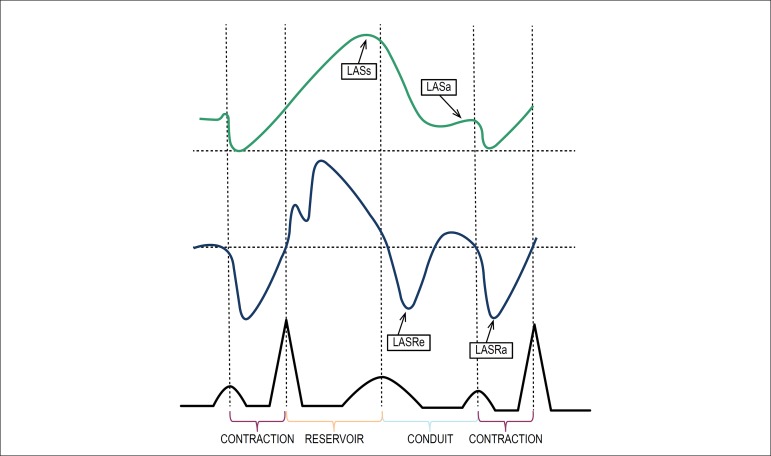
Left atrial phasic functions and their relationship with the cardiac cycle. Strain and strain rate curves are shown. During left ventricular (LV) systole and isovolumetric relaxation, left atrium (LA) works as a distensible reservoir accommodating blood flow from the pulmonary veins. During early LV diastole, LA behaves as a conduit that starts with mitral valve opening and terminates before LA contraction, allowing passive emptying during early ventricular diastole and diastasis. Finally, at end-diastole, LA acts as a muscular pump contributing to LV filling with active atrial contraction. (LASs: left atrial systolic strain; LASa: left atrial diastolic strain; LASRe: early left atrial strain rate; LASRa: late left atrial strain rate).

Strain parameters were obtained during ventricular systole (LASs - LA systolic strain) and diastole (LASa - LA diastolic strain), and strainR parameters were obtained during early (LASRe - LA strainR early) and late (LASRa - LA strainR late) ventricular diastole (Figure 1) in 2 standard echo-views (apical 4- and 2-chamber views), using speckle-tracking echocardiography to avoid the angle-dependence of DTI.^6,12^

In addition, 30 healthy individuals were analyzed following the same protocol in order to have a reference population for LA mechanics.

Two experienced observers carried out the measurements in both populations in different times, in order to determine intra- and inter-observer variability. Intra-observer variability was calculated with measurements of the same rater in different moments including random samples of either patients or healthy controls. The same 2D echocardiographic loops of random samples of both patients and healthy controls were used for inter-observer variability.

All images were digitally stored for offline analysis.

### Definition of AF recurrence

AF recurrence was defined as presence of AF at any electrocardiogram or during at least 30 seconds in Holter monitoring.

### Statistical analyses

Descriptive analyses were performed to explore study population characteristics. Categorical variables were reported as frequencies, and continuous variables with normal distribution were reported as mean ± SD. Median and interquartile range were used in cases of non-normality. Normal distribution of continuous variables was studied using Kolmogorv-Smirnov test.

Differences among cohorts were analyzed using Chi-square test for categorical variables (or Fisher´s exact test when the comparison group was < 30 individuals), and Student-*t*-test (or Mann-Whitney test if the comparison group was < 30 and in case of non-normal distribution) for the numerical ones.

Kaplan-Meier method was used for describing event free survival (AF) over time; the median was used as cutoff value to compare quantitative variables and differences between groups were investigated with the log-rank test. Those variables with p value < 0.15 were included for multivariate analyses using a Cox proportional hazard model.

Significance was considered with a p value of < 0.05.

Statistical analyses were performed using SPSS (Statistical Program for the Social Sciences [SPSS Inc., Chicago, USA]) version 15.0.

Intra- and inter-observer agreements in the speckle-tracking measurements were studied by regression analyses and calculation of the intraclass correlation coefficient. Bland Altman plots, combined with calculation of 95% limits of agreement were also generated. For this analysis MedCalc Statistical Software version 15.6.1 (MedCalc Software bvba, Ostend, Belgium) was employed.

## Results

AF recurrence was identified in 9 out of 53 cases in the immediate post-surgery period (3 months). These 9 patients were excluded for subsequent follow-up.

The 44 patients with sustained SR after 3 months were included in the echocardiographic and rhythm follow-up. Baseline characteristics of this series are shown in Table 1.

**Table 1:** Baseline characteristics of patients who maintained sinus rhythm in the immediate post-surgery period (3 months) (n = 44)

Characteristics	
Age (years)	69 ± 9
Female gender, n (%)	32 (73%)
Mitral surgery, n (%)	36 (82%)
Aortic surgery, n (%)	16 (37%)
Tricuspid intervention, n (%)	13 (29.5%)
Cryoablation, n (%)	36 (82%)
Antiarrhythmic treatment at discharge, n (%)	13 (29.5%)
ACE inhibitors at discharge, n (%)	21 (48%)
AF duration > 1 year before surgery, n (%)	26 (59%)
LA biplane volume (mL/m^2^)	68 ± 22
AP LA diameter (mm/m^2^)	28.9 ± 5
LVEF (%)	63 ± 12

ACE: angiotensin converting enzyme; AF: atrial fibrillation; AP: anteroposterior; LA: left atrium; LVEF: left ventricular ejection fraction.

The majority of the population underwent mitral surgery (28 patients, 63.6%), 8 (18.2%) mitral and aortic, and only 8 required exclusively aortic intervention. Mitral valve surgery included 34 prosthetic replacement procedures (26 mechanical and 8 biological), and two mitral valve repair surgeries. Valve replacement was the procedure employed in all patients with aortic disease (12 mechanical and 4 biological). There were 13 (29.5%) tricuspid annuloplasties using Carpentier-Edwards ring in all cases.

Overall, the study population showed preserved left ventricular ejection fraction (LVEF) and severely dilated LA. These patients were predominantly women with a mean age of 69 ± 9 years old (y.o.). Treatment at discharge included amiodarone in 30% of patients and angiotensin-converting-enzyme (ACE) inhibitors in 48% of patients.

After a mean follow up of 17 ± 2 months, 13 new post-operative cases of AF were identified.

**Figure 2:**
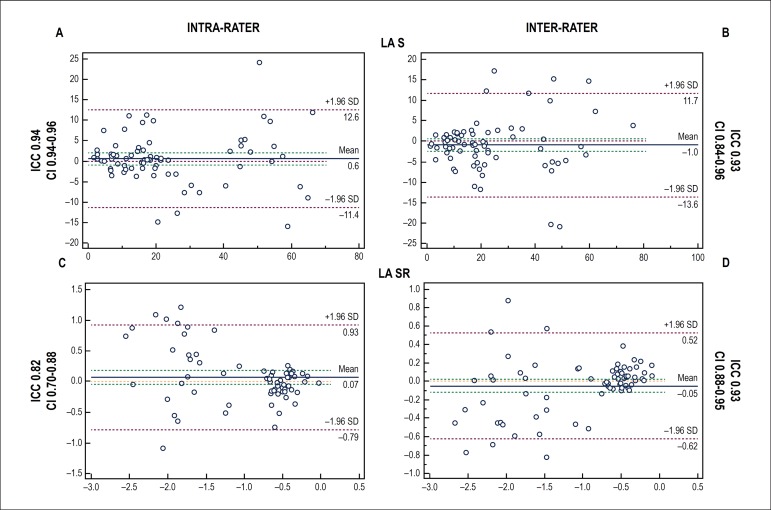
Intraclass correlation coefficient calculated (ICC) and Bland Altman difference plot combined with calculation of 95% limits of agreement (CI) of the intra-rater (A,C) and inter-rater (B,D) agreement of LA strain (LA S) and LA strain rate (LA SR)

Myocardial deformation parameters (strain and strainR) for assessing LA mechanical function after SA were obtained from 1,245 left atrial segments that were correctly analyzed (71% of possible). On average, 15.5% and 19.4% of 24 potential segments were analyzed per patient and per control, respectively. LA mechanical function (strain and strainR) was significantly worse in all patients than in normal population, independently of SR maintenance (Table 2, Figure 2).

**Table 2:** Strain and strain rate parameters in the post-surgery group versus healthy individuals (Median and Interquatile Range were used because non-normal distribution of variables). P values were calculated with Mann-Whitney test

	LASs Median (P_25_-P_75_)	LASa Median (P_25_-P_75_)	LASRe Median (P_25_-P_75_)	LASRa Median (P_25_-P_75_)
Post-surgery group	16.9 (14.1-20.6)	5.9 (4.5-7)	-0.55 (-0.45- -0.67)	-0.41 (-0.56- -0.25)
Control group	42.5 (36.3-48.8)	13.1 (11.6-16.2)	-1.83 (-1.4- -2)	-1.6 (-1.8- -1.4)
p	< 0.001	< 0.001	< 0.001	< 0.001

LASe: LA systolic strain; LASa: LA diastolic strain; LASRe: LA early strain rate; LASRa: LA late strain rate

**Figure 3:**
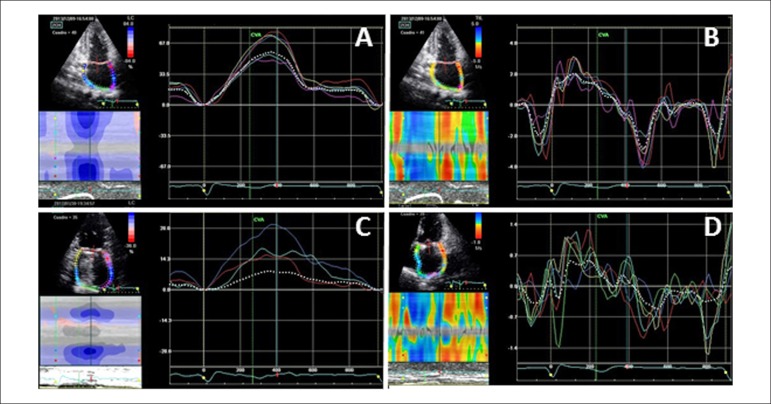
Strain and strain rate curves in a healthy individual (A and B, respectively) and in a post-surgery patient (C and D, respectively).

As showed in Figure 3, intraclass correlation coefficient was always > 0.80, that represents good to excellent reliability and reproducibility of measurements.^13^

The univariate analyses showed a trend of AF recurrence related to age, mitral surgery, cryoablation and LA biplane volume (Table 3). Patients with mitral valve intervention and cryoablation were younger (66.6 ± 8.4 vs 73.6 ± 9.1 y.o.; p = 0.041). As patients treated with cryoablation were the same as those with mitral intervention, cryoablation was not included in further analyses to avoid collinearity in the multivariate analysis. No association was found between deformation parameters and AF recurrence.

**Table 3:** Univariate analysis

	AF recurrence n = 13	SR maintenance n = 31	p (Univariate)
LA biplane volume (ml/m^2^)	76.4 ± 25.5	63.7 ± 19.2	0.059
Age (years)	71.5 ± 7	66 ± 9	0.055
Mitral surgery	9 (25%)	27 (75%)	0.087
Antiarrhythmic treatment at discharge	3 (23%)	10 (77%)	0.498
ACE inhibitors at discharge	6 (28.6%)	15 (71.4%)	0.454
AF duration > 1 year before surgery	9 (34.6%)	17 (65.4%)	0.748
LASs*	14.1 (13.1-20.1)	17.2 (15.4-21.4)	0.961
LASa*	5.6 (3.3-6.3)	5.9 (4.7-7.4)	0.385
LASRe*	-0.5 (-0.45- -0.67)	-0.5 (-0.45- -0.67)	0.965
LASRa*	-0.4 (-0.25- -0.59)	-0.4 (-0.25- -0.58)	0.961

P values were calculated with the use of the Mann-Whitney or Fisher´s exact tests.

(*) Variables with non-normal distribution (median and intercuartile range [P_25_-P_75_]). ACE: angiotensin converting enzyme; AF: atrial fibrillation; LA: left atrium; LASs: LA systolic strain; LASa: LA diastolic strain; LASRe: early LA strain rate; LASRa: late LA strain rate.

**Figure 4:**
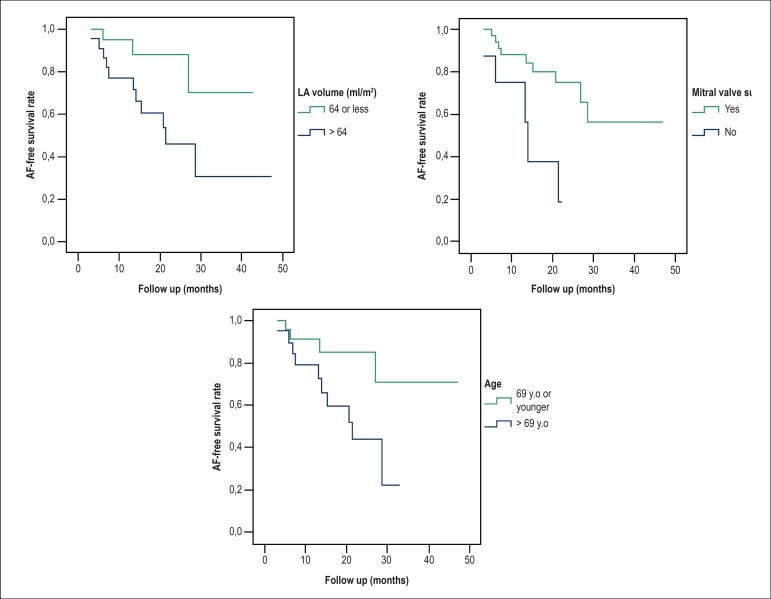
Log-rank test for comparison of Kaplan-Meier curves according to mitral valve surgery (p = 0.006), age > 69 years old (p = 0.027) and left atrial volume > 64 ml/m^2^ (p = 0.030). AF: atrial fibrillation; LA: left atrial; y.o.: years old

As can be seen in Figure 4, employing the univariate log rank test, AF recurrence seems to be associated with: larger LA volume (p = 0.030), older age (p = 0.027), and inversely, with mitral valve intervention (p = 0.006).

A Cox proportional hazard model was built to explore potential sources of confusion and interactions. After SA of AF, LA volume was the only parameter associated with sustained SR (p = 0.028). Mitral surgery (p = 0.056) and age (p = 0.412) were not significantly associated with SR maintenance in the multivariate analyses.

## Discussion

AF is the most common arrhythmia in general population and is even more common in VHD patients. This arrhythmia is cause of symptoms, hospital admissions, adverse events (systemic embolisms, side effects of antiarrhythmic drugs, etc.) and therefore, has a high impact in survival and quality of life. In addition, the presence of AF determines the necessity for antithrombotic therapy, and even the selection of the type of prosthesis.^1,2^

This study was conducted in patients who would otherwise have been chosen to heart rate control. Due to the scarcity of data about this treatment in “pure” VHD series, the current study may provide novel insights in this clinical setting. We found that after 28 months, 50% of VHD patients with initial successful ablation remained in SR.

Veasey et al.^14^ reported rates of SR of 74% in paroxysmal AF and 51% in persistent AF; nevertheless, the mean follow-up time was only 6 months, and, 39% of these patients had exclusively coronary artery bypass surgery. Similar results were found by Gaynor et al.^15^ and Budera et al.;^16^ 71% of patients had sustained SR after 6 months and 53.2% after one year respectively, but, these series included patients with lone AF surgery and revascularization for ischemic heart disease. Beukema et al.^17^ reported one of the largest series including 285 patients with structural heart disease, finding that SR was present in 57.1% of patients after 5 years of follow- up; however, this study does not state the rate of patients with VHD.

The consensus statement of the American Society of Echocardiography and the European Association of Echocardiography suggests that LA mechanics can be assessed after AF to predict the maintenance of sinus rhythm and after percutaneous atrial septal defect repair. In addition, LA mechanics may offer suitable parameters to identify patients at risk for LA regional failure or arrhythmias or to assess LA characteristics in patients with LA dilatation of undetermined cause.^18^ LA strain has also been used to predict post-operative AF after mitral valve intervention.^19^ However, there are no previous data describing LA mechanics after concomitant AF surgical ablation in VHD patients or series aiming to obtain the relationship between recurrence and atrial mechanics in this group of patients.

**Figure 5:**
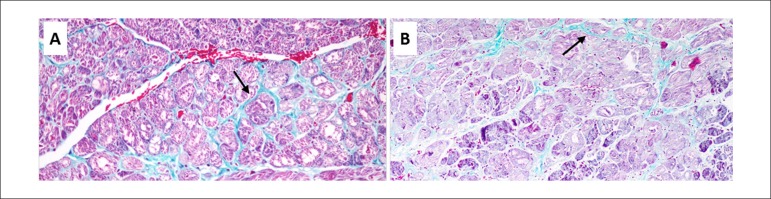
Masson's Trichrome. Collagen fibers are stained blue. A) AF recurrence 6 days after ablation. Abundant atrial fibrosis (arrow) of perivascular predominance. B) Sinus rhythm maintenance during follow-up. Mild atrial fibrosis (arrow).

We speculate that the lack of association of LA strain and strainR parameters with AF postoperative recurrence may be explained by severe atrial dilation with extensive areas of fibrosis before surgery in both responders and non-responders to AF ablative techniques. Very large areas of atrial fibrosis may result in an important decrease in atrial mechanics, as shown by deformation parameters of patients included in this study in comparison with healthy individuals. Comparisons between responders and non-responders to AF are limited by the very low parameters of atrial deformation in all patients, which affects the sensitivity of deformation parameters to predict AF recurrences. However, the more fibrosis, the less likely it is to maintain SR. For illustrating this hypothesis, in Figure 5, we compare the anatomopathological characteristics of a patient with more extensive fibrosis and AF recurrence (Figure 5A) with another in SR during follow-up (Figure 5B). Larger studies and inclusion of LA tissue sampling should be necessary to demonstrate this hypothesis.

In accordance with prior reports,^20^ in the current study, larger LA was associated with AF recurrence, suggesting that patients who could benefit more from this technique are those with LA volume < 64 ml/m^2^. Another novel contribution of the series presented here, is that despite the fact that LA diameter has been traditionally considered one of the major inclusion criteria for candidate selection, only LA volume appears as a predictor of AF recurrence. To the best of our knowledge, the prognostic value of LA biplane volume to predict recurrences after AF cryoablation in VHD patients has not been previously reported and may contribute to better selection of candidates with VHD.

The suppression of AF was most successful in patients undergoing mitral valve surgery, patients in whom cryoablation was systematically used. Patients who underwent aortic valve surgery and AF ablation with HIFU-Epicor had significantly lower rate of SR maintenance. In prior publications, the success rate of Epicor system was also lower.^21^ Endocardial approach (used in cryoablation) has shown higher success rates in comparison with the more superficial epicardial approach (HIFU-Epicor). However, according to other authors, this difference may not be due uniquely to the lower efficiency of the ablation system employed, and they speculate whether the underlying heart disease may also influence outcome, because it is well known, that isolated mitral valve surgery (without additional AF ablation) has a significant beneficial effect on spontaneous conversion to SR.^5,21^

Antiarrhythmic management is important in patients with recurrent AF in the post-operative period in improving results of SA.^4^ However, in our study, no association was found between antiarrhythmic treatment and SR maintenance. We could not infer from our data whether this finding was due to the small number of patients was discharged with amiodarone, or if amiodarone is not effective for SR maintenance in these patients.

In the univariate analyses, age was associated with the recurrence of AF, however, this relationship was not observed after multivariate testing. It appears, therefore, that age is a confounding variable, since patients undergoing both mitral valve intervention and cryoablation are significantly younger.

It is well known that AF is associated with LA myocardial remodeling and ultra-structural changes, including fibrosis and accumulation of extracellular matrix - effects that may predispose to the formation of zones of slow conduction, which promote re-entry.^22^ ACE-inhibitors are thought to reduce atrial dilatation, dysfunction, and fibrosis, which may reduce the propensity for developing AF.^23^ In some studies, after catheter ablation, there is a trend towards fewer AF recurrences in patients treated with ACE-inhibitors, however, the efficacy of this treatment in routine clinical practice remains unknown.^24^ In the present study, ACE-inhibitors were used in a substantial proportion of patients (48%), but they were not found to be effective enough for preventing AF recurrence.

As it has been demonstrated in previous studies,^25^ AF surgical ablation is a safe procedure without increasing total surgical time, in comparison with the traditional Cox-Maze procedure, which has an elevated success rate but significantly increases intraoperative time. In our series we have not found major complications related to this technique.

### Limitations of the study

Despite the systematic use of 24 hour Holter monitoring in the present series, silent AF remains an important issue in the post-operative follow-up of this type of patients. A major limitation of studies about AF treatment is that the burden of arrhythmia cannot be a reliable determinant unless an implantable device is used. It is also difficult to make a proper comparison with other studies in the absence of universal criteria for defining AF recurrences.

Results on the antiarrhythmic treatment should be interpreted with caution, since treatment with amiodarone (which was not uniformly employed across patients) could affect the success of the AF ablation technique. When antiarrhythmic drugs were forced into the multivariate model, recurrence predictors remained unchanged.

This study was carried out for a limited time period, with a relatively small sample size and in a single tertiary center. Multicenter studies and larger number of patients will be needed in the future to obtain more evidences about efficacy and safety of this technique in VHD patients.

## Conclusions

Left atrial volume was larger in patients with AF recurrence, and emerges as the main predictor of recurrences improving candidate selection for this therapy; however, no differences were found regarding myocardial deformation parameters. Despite electrical maintenance of SR, left atrium mechanics did not recover after AF ablation performed during VHD surgery.
